# Hospital Meetings

**Published:** 1904-05-28

**Authors:** 


### HOSPITAL MEETINGS.

### PADDINGTON GREEN CHILDREN'S HOSPITAL.

PADDINGTON GREEN CHILDREN'S HOSPITAL celebrated its coming of age on Thursday, May 19th, when the annual meeting was held. The proceedings opened with a reception and afternoon tea in the vestibule, which was prettily decorated with flowers and palms; and the board-room, in which the meeting took place, was well filled, a large number of those present being ladies. The chair was taken by Mr. Douglas Owen, who has just been appointed to the chairmanship of the committee in place of Mr. George Hanbury. The Chairman was supported by the Hon. W. H. Goschen, Mr. Robert Hanbury, and others.

The report for 1903 having been read, its adoption was moved from the chair. Mr. Owen referred in terms of regret to the death of Mr. George Chance, who, as a member of the Hospital Board for more than 20 years, had always had the welfare of the Institution deeply at heart. Alluding next to the resignation, owing to the state of his health, of Mr. George Hanbury, the Chairman said his loss was a calamity to the hospital which the committee could only promise to do their best to repair. The two new members of the committee were Mr. Cecil Sebag-Montefiore and Mr. Robert Ogilvie. It was Mr. George Hanbury who, in 1881, was the prime mover in converting a very useful little charity, known as the North-West London Free Dispensary for Sick Children, into the Paddington Green Children's Hospital, at a cost of some £8,000. Two years later an out-patient department was provided at an outlay of £6,000. Later still, the entire structure had to be demolished on account of outbreaks of diphtheria, and in 1885 the present building was opened, having cost close upon £15,000. In the following year an addition was made at a cost of £4,000. In 1902 the convalescent home at Slough was opened, at a cost of £2,000, the number of beds being now 46 at the hospital and 16 at the home. Throughout the whole of the hospital's career Mr. George Hanbury had been the moving spirit, and it was hardly possible te estimate the indebtedness of the committee to him for his constant devotion to its interests. A resolution would subsequently be moved on the subject, but before leaving it, he wished to refer to a notice sent out by himself, headed "Not an Appeal," in which he asked for the support of the friends of the hospital for himself as the new chairman. If the committee, under its new chairman, were as successful, and as free from mistakes, as under Mr. Hanbury, there need be no fear for the future. Turning to the report, the chairman said an important event of the year was the concert at Queen's Hall, under the patronage of H.R.H. the Princess of Wales, who was present on the occasion. This special effort resulted in the sum of £611 19s. 3d. for the funds of the hospital and convalescent home. Their thanks were due to all concerned, as well as to other friends, through whose kind efforts other entertain ments were given during the year. These undertakings could not be measured by their immediate results in money, for they exerted a wider influence, in the interest which they stimulated, and they were almost sure to bring new friends to the hospital. The annual expenses of the hospital were now, in round figures, about £5,000, those of the home being about £1,000. To meet this, there was a certain assured income, leaving, however, a shortage of about £2,000, and it was to meet this that entertainments were necessary. His notice "Not an Appeal" had brought in £50, some kind-hearted people having regarded it as an appeal, and there were further promises. These sums, however, did not bear much relation to the debt of £2,600 which the committee had to meet. They were not going to be frightened by its magnitude; he claimed that past events justified them in optimism. He hoped no one would think there had been undue extravagance in the decorations; for these they were indebted to Miss Strudwick, who most kindly supplied not only the annual meeting, but the wards all the year round with flowers. The convalescent home had proved of such value that, at all costs, the committee were determined to continue it. All the beds were available throughout the year, and 139 children were admitted, the average length of stay being 38 days. The transfer of these cases to the home from the wards at an early stage of convalescence not only materially assisted in the more rapid and complete recovery of the patients, but also enabled many more acute cases to be admitted to the hospital. Indeed, the various departments had worked throughout the year at quite exceptional pressure, the number of patients being the largest on record, viz., 17,460, of whom 654 were in-patients.

The Hon. W. H. Goschen, in seconding the motion, described the wards as veritable children's palaces. He was glad to have the opportunity of supporting their new Chairmon, whom he knew to be a man of restless energy, great business capacity, and great human kindness. He appealed to those who had known what it was to receive back a sick child of their own, thanks to the skill of doctors and nurses, to come forward and endow cots in this admirable hospital for children.

The following resolution was then proposed by the Chairman, seconded by Dr. Guthrie, and passed unanimously:—"That the Governors and supporters of the Paddington Green Children's Hospital in annual meeting assembled desire to express their profound regret at the resignation of Mr. George Hanbury of his Chairmanship of the Committee of Management. They desire further to offer him their cordial and hearty thanks for his devoted services to the hospital since the year 1881, when, as the outcome of his unselfish efforts, the Charity was founded, and to express their gratification that his resignation of the Chairman's duties will not involve either his vacation of the post of Treasurer or any diminution of his interest in the hospital."

Other speakers were Mr. Robert Ogilvie, Mr. Robert Hanbury, Dr. Alexander Morrison, Mr. Wakefield, the Rev. Conrad Noel, Lieut.-General Lowry, and Mr. Hunter Todd. The usual elections having taken place, and votes of thanks passed to the various officers, including one warmly appreciative of the work of the medical staff, and of the nursing staff under the matron, Miss Pinchard, the meeting closed with thanks to the Chairman.

### THE HOSPITAL FOR WOMEN, SOHO.

The sixty-first annual Court of Governors of this hospital was held on Friday, May 20th, when the chair was occupied by the President, the Earl of Shaftesbury. There was a good attendance, many ladies being present.

The report for 1903 having been read, the Chairman said it was, on the whole, as satisfactory as any report issued by the hospital during the last few years. The average number of beds occupied daily throughout the year was the highest yet attained, viz., 55, the average residence of each patient being 23 days. The subscribers would appreciate the importance of the reduction which had been made in the average weekly cost of each in-patient, namely, from £2 8s. 9d. in 1902 to £2 1s. 9d. in the year under review, and of each out-patient from 3s. 6d. to 2s. There were 4,444 new out-patients, with 17,103 attendances. Eighty-eight patients were sent to convalescent homes, and £30 was expended, through the agency of the Reardon and Sturge Samaritan Fund, in the purchase of surgical appliances. Their best thanks were due to Her Royal Highness Princess Christian, through whose kind initiative the Lady Mayoress had been able to establish successfully a Ladies' Association, by the aid of which £400 were added to the funds of the hospital. He was sorry to say there was a deficit of £426 13s. 2d. on the year's expenses, the income being £6,198 1s. 11d., including legacies amounting to £339 11s. 9d., and the expenditure £6,624 15s. 1d., but as a set-off against this a sum of £1,000 was paid in reduction of the mortgage debt.

With reference to the important question of reconstruction or removal to another site the committee, having regard to the fact that appeals were now being made to the charitable public for very large sums for the removal or extension of older and larger hospitals, felt the difficulty of taking any immediate step. An opportunity having occurred, however, of acquiring additional freehold property immediately adjoining the hospital premises, they had purchased No. 3 Frith Street, which would be of the greatest value when funds were available for the extension or rebuilding of the hospital. For the purpose they raised a loan of £2,500 which had increased the debt secured by mortgage of the hospital buildings to £7,500. Notwithstanding the many difficulties with which the committee had to contend he thought it must be recognised that the hospital was doing very excellent work, and he wished especially to draw attention to the low death-rate of the patients, most of whom had to undergo very serious operations. It compared favourably with that prevailing at the most modern and up-to-date institutions. In conclusion, the committee desired to acknowledge the financial assistance rendered by the numerous friends of the hospital, and he ventured to hope that a larger number still would come forward to help this old-established and most useful charity.

Mr. Victor Williamson, in seconding the adoption of the report, agreed with the chairman as to the wisdom of the committee's recommendation. It had been arrived at after very careful consideration and inquiries, in the course of which a site at Stamford Hill had been inspected. The distance, however, was a fatal objection to what appeared to be an otherwise ideal site, and the idea of removal there was considered impracticable. He desired to emphasise the fact that the excellence of the work of the hospital was practically testified to by the Council of King Edward's Hospital Fund, from whom a grant of £1,100 had been received, to maintain the 18 beds re-opened by their aid. The committee also gratefully acknowledged grants of £130 and £109 7s. from the Hospital Sunday and Saturday Funds respectively.

The election of new Governors, committee, and other officers having taken place, the meeting concluded with a vote of thanks to the chairman.

